# COVID‐19 Waves and Cardiac Health: An Investigative Analysis of Creatine Phosphokinase Levels and Troponin Status Using Machine Learning

**DOI:** 10.1002/hsr2.71501

**Published:** 2025-11-17

**Authors:** Amirhossein Shahpar, Nazanin Zeinali Nezhad, Niloofar Farsiu, Marzieh Charostad, Masoud Rezaei, Faranak Salajegheh, Mohammad Pardeshenas, Seyedeh Mahdieh Khoshnazar, Mohsen Nakhaie

**Affiliations:** ^1^ Gastroenterology and Hepatology Research Center, Institute of Basic and Clinical Physiology Sciences Kerman University of Medical Sciences Kerman Iran; ^2^ Physiology Research Center Kerman University of Medical Sciences Kerman Iran; ^3^ Department of Biology, Faculty of Science Yazd University Yazd Iran; ^4^ Research Center for Hydatid Disease in Iran Kerman University of Medical Sciences Kerman Iran; ^5^ Research Center of Tropical and Infectious Diseases Kerman University of Medical Sciences Kerman Iran; ^6^ Department of Microbiology, School of Medicine Kerman University of Medical Sciences Kerman Iran; ^7^ Student Research Committee Kerman University of Medical Sciences Kerman Iran; ^8^ Clinical Research Development Unit, Afzalipour Hospital Kerman University of Medical Sciences Kerman Iran

**Keywords:** biomarker, cardiac health, COVID‐19, creatine phosphokinase, machine learning modeling

## Abstract

**Background:**

This study explores the correlation between creatine phosphokinase (CPK) levels and cardiac troponin status with eight waves of COVID‐19 and identifies the most significant biomarker for assessing disease severity.

**Methods:**

Participants were selected based on confirmed COVID‐19 diagnoses using RT‐PCR testing. Machine learning modeling with the PyCaret autoML library established a benchmark for classification models using variables such as age, gender, serum troponin, CPK, and COVID‐19 waves. Rigorous evaluation metrics were employed to assess model performance.

**Results:**

The analysis included 1975 COVID‐19 patients. Patient demographics showed a shift in age and gender distribution across different waves, with later waves characterized by younger patients and a greater proportion of females. Mortality rates varied, peaking at 34.5% in the third wave and dropping to 0% in the eighth wave. CPK levels differed significantly among waves, with the third wave having the highest levels and later waves showing the lowest levels. However, troponin positivity rates did not differ significantly among waves. An extra trees classifier model achieved an overall accuracy, micro‐average area under curve (AUC), sensitivity, and specificity of 0.79, 0.65, 0.79, and 0.89, respectively. CPK was identified as the most important predictor of patient outcome, followed by COVID‐19 wave, age, and gender, while troponin status had the least importance.

**Conclusion:**

These findings shed light on the potential relationship between CPK, troponin, and different waves of COVID‐19 and their impact on disease severity. This understanding could significantly contribute to future research and clinical practices, aiding in the management and mitigation of COVID‐19's cardiac implications.

## 1 Introduction

COVID‐19 is a highly contagious respiratory illness caused by a novel coronavirus. The virus was initially identified in Wuhan, China, in December 2019 and has rapidly spread worldwide [1]. As of March 2024, the World Health Organization (WHO) reports over 700 million confirmed COVID‐19 cases and nearly seven million deaths worldwide [2]. These numbers suggest a global case fatality rate (CFR) of roughly 1%. However, estimates of the CFR can vary considerably depending on factors such as population demographics, testing coverage, and reporting accuracy. For instance, Our World in Data cites slightly higher figures—around 772 million cases and 7.1 million deaths. Some researchers, including Ioannidis (2021), have suggested that the true fatality rate may be much lower in many contexts, with estimates as low as 0.15%, especially in younger populations or where widespread testing was available [1]. Overall, published estimates of the CFR range from about 0.1%–0.3% in large‐scale population studies to around 1% when calculated from reported case and death counts [1, 2].

Following SARS‐CoV‐2 infection, clinical manifestations vary significantly among individuals. Our data shows that while some patients remain asymptomatic or experience mild symptoms, others may develop more severe disease [2]. SARS‐CoV‐2 enters human cells through multiple pathways. While angiotensin‐converting enzyme 2 (ACE2) is the primary receptor, recent research has identified several ACE2‐independent alternative receptors involved in viral entry. These include CD147, AXL, CD209L/L‐SIGN/CLEC4M, CD209/DC‐SIGN/CLEC4L, and others [3]. These receptors are found in various organs including the small intestine, kidney, gallbladder, heart, and liver, contributing to the virus's multi‐organ tropism [4]. ACE2 is crucial for the process of cellular entry and is present in many mammalian species, making infection possible [5].

Respiratory failure is considered to be the primary cause of mortality in individuals affected by COVID‐19, especially among older adults. However, there are also some patients who may suffer from cardiovascular‐related conditions such as congestive heart failure (CHF) and brain medullary cardiorespiratory dysfunction, which can contribute to their overall mortality rates [6]. COVID‐19 can damage the heart tissue through three distinct mechanisms. Firstly, the acute hyper‐inflammatory response triggered by the infection can lead to the formation of blood clots that obstruct arteries. Second, the infection of the heart muscle itself, along with the subsequent inflammatory response, can result in tissue damage. Lastly, infection can occur in the vascular endothelium and heart pericytes, which is commonly referred to as vasculitis [7].

During the initial investigations conducted in Wuhan, it was found that heart failure was a prevalent complication affecting about 24% of all patients [7]. Therefore, it can be beneficial to assess heart function using various examinations to manage the condition. Several examinations have been conducted to evaluate the functionality of the heart, including the assessment of creatine phosphokinase (CPK) levels and cardiac troponins I [8, 9]. It is common for hospitalized patients to experience a significant increase in serum CPK level, which can be attributed to either skeletal muscle or myocardial injury. In contrast, cardiac troponins are released from infected myocardium in a time‐dependent manner. They can be detected in the bloodstream about 4–6 h after the onset of myocardial infarction, reach their highest concentration at around 16–18 h, and continue to be detectable at elevated levels for at least 7 days [9].

The heart plays a vital role in the human body, and it's crucial to use all available treatment methods to ensure its optimal function when treating COVID‐19. Iran has experienced eight waves of COVID‐19 caused by different variants of SARS‐CoV‐2 until the time of data collection for this investigation. This article provides a comprehensive and comparative analysis of CPK levels and troponin status throughout the eight waves of the COVID‐19 pandemic. The study aims to uncover potential correlations and patterns between these biomarkers and the severity of the disease, which can contribute to a broader understanding of COVID‐19's pathophysiology and impact on cardiac health. The results may help guide future research and inform clinical practices for managing and mitigating the cardiac implications of COVID‐19.

## 2 Materials and Methods

### 2.1 Participant Selection and Ethical Approval

The study involved 1975 adult patients who were admitted to Shahid Sadoughi Hospital in Yazd, Iran, between February, 2020 and May 20, 2023. All participants were diagnosed with COVID‐19 using RT‐PCR in accordance with the WHO criteria. Ethical approval (IR.SSU.REC.1400.207) was obtained from the Research Ethics Committee of Shahid‐Sadoughi University of Medical Sciences, and informed consent was taken from each participant.

### 2.2 Study Protocol and Data Extraction

The study analyzed data from eight different waves of the COVID‐19 pandemic. Information was extracted from the health information systems of eligible patients who had a confirmed RT‐PCR diagnosis based on WHO criteria. Data collection occurred daily throughout the study period as patients were admitted. Biomarker measurement (CPK and troponin) was obtained during routine clinical care at hospital admission, following standardized hospital protocols. Patients with comorbidities, missing data on evaluated biomarkers, or an alternative diagnosis were excluded from the study. Each wave included a random sample of 300 COVID‐19 patients, except for the sixth (252), seventh (136), and eighth (87) waves. Patients were grouped into three categories based on the severity of their disease during hospitalization: partial recovery, full recovery, and nonsurvivor. Demographic information, pandemic wave data, clinical outcome data, and biomarker data (CPK, troponin) were extracted from the health information systems. To ensure accuracy and reliability, two independent and trained investigators, who were unaware of each other's findings, extracted information from the health information systems. Any discrepancies in chart abstraction were resolved through discussion.

#### 2.2.1 Biomarker Measurement Methodology

CPK levels were measured quantitatively and reported as continuous values (median with interquartile range). Cardiac Troponin I (cTnI) was assessed qualitatively as positive/negative according to standard hospital protocols, with results reported as percentages of positive tests. We acknowledge that these different measurement scales limit direct comparisons between the two biomarkers.

### 2.3 Machine Learning Modeling

All machine learning models were built and evaluated using the Python programming language (version 3.8). PyCaret autoML library (https://pycaret.org/), a high‐level wrapper for the scikit‐learn library, was used for model training and evaluation. The library was used to establish a benchmark for all accessible classification models. The variables taken into account for this model included the outcome variable, which was set as the target variable, and several independent variables: age, gender, serum troponin, CPK, and the different waves of COVID‐19. These variables were chosen based on their potential influence on the outcome of COVID‐19, as suggested by prior research. The model selection process focused on accuracy, and the model demonstrating the highest accuracy was selected for further analysis.

### 2.4 Model Training and Evaluation

The data were divided into train (70%) and test (30%) sets, and 10‐fold cross‐validation was used for the training phase. The evaluation of the model's performance was accomplished using a variety of evaluation metrics. The feature importance plot was employed to assess the impact of the variables on the outcome. The evaluation metrics used in the study included accuracy, F1 score, micro‐average area under curve (AUC), positive predictive value (PPV), sensitivity, and specificity. Also, the SMOTE‐Tomek approach was utilized to handle imbalanced sampling in the data.

### 2.5 Statistical Analysis

Continuous variables that had a normal distribution were presented as the mean along with the standard deviation. On the other hand, continuous variables that did not follow a normal distribution were reported as medians with a range of 25th to 75th percentile. Before analysis, all the data were tested for normality of distribution and equality of standard deviations.

The Mann‐Whitney *U* and Kruskal‐Wallis tests were used to evaluate continuous variables that did not follow a normal distribution. When necessary, adjustments were made for multiple comparisons to address the issue of Type I error. The Chi‐Square test was used to analyze the association between categorical variables. The data analysis was conducted using IBM SPSS Statistics version 27 for Windows (SPSS Inc., Chicago, Illinois, USA). A *p*‐value of less than 0.05 was considered significant.

### 2.6 Ethical Considerations

Ethical guidelines for human research were strictly followed, including obtaining ethical approval as well as taking measures to protect participant privacy and confidentiality.

## 3 Results

### 3.1 Age Distribution and Pandemic Progression: Unveiling the Evolving Demographic Profile

Our research involved 1975 individuals who were diagnosed with COVID‐19. The study included participants between the ages of 1 and 116, with a median age of 60 and an interquartile range of 43–74 years. The average age of patients was highest during the third wave (61.36 ± 20.97 years) and lowest during the seventh wave (48.36 ± 24.31 years). This suggests a change in the age distribution of COVID‐19 patients over time. The shifts were statistically significant (*p*‐value < 0.001), indicating that the disease's demographic profile is evolving (Table 1).

**Table 1:** COVID‐19 patient characteristics and biomarkers findings across eight waves.

	Total	Age		Gender Number (%)		CPK		Positive troponin
Wave	Number (%)	Mean (SD)	Median (IQR)	Male	Female	Mean (SD)	Median (IQR)	Number (%)
1st	300	60.80 (18.05)	61.0 (21.75)	165 (55.0)	135 (45.0)	148.43 (171.16)	94.5 (113.00)	11 (3.7)
2nd	300	59.77 (18.34)	61.0 (25.0)	171 (57.0)	129 (43.0)	251.14 (420.75)	120.0 (175.00)	6 (2.0)
3rd	300	61.36 (20.97)	68.0 (33.75)	173 (57.7)	127 (42.3)	249.17 (500.30)	131.5 (172.25)	8 (2.7)
4th	300	54.11 (18.20)	53.0 (33.75)	156 (52.0)	144 (48.0)	201.92 (269.70)	106.5 (156.00)	4 (1.3)
5th	300	57.48 (16.38)	57.0 (26.75)	162 (54.0)	138 (46.0)	254.75 (561.52)	110.5 (151.75)	2 (0.7)
6th	252	59.76 (20.77)	62.5 (37.00)	119 (47.2)	133 (52.8)	110.03 (117.69)	73.0 (74.00)	4 (1.6)
7th	136	48.36 (24.31)	48.0 (37.25)	58 (42.6)	78 (57.4)	125.67 (73.44)	124.0 (101.25)	4 (2.9)
8th	87	48.37 (28.10)	46.0 (38)	42 (48.3)	45 (51.7)	53.13 (10.05)	50.0 (15.00)	0 (0.0)
*p*‐value		< 0.001		< 0.001		< 0.001		0.135

### 3.2 Variation in Gender Distribution: Exploring the Dynamic Shift Across Pandemic Waves

Out of all patients, 53% were males (1046 individuals), and 47% were females (929 individuals), giving a male‐to‐female ratio of 1.12. Interestingly, during the early stages of the pandemic, there was a higher prevalence of males, with a male‐to‐female ratio of 1.36 in the third wave. However, this trend gradually changed in the subsequent waves, and in the seventh wave, there was a higher prevalence of females with a male‐to‐female ratio of 0.74. This significant variation in gender distribution across different waves suggests that there may be biological or behavioral factors that influence gender susceptibility to COVID‐19 (*p*‐value < 0.001) (Table 1).

### 3.3 Mortality Rates: Fluctuating Patterns and Wave‐Specific Insights

**Figure 1:**
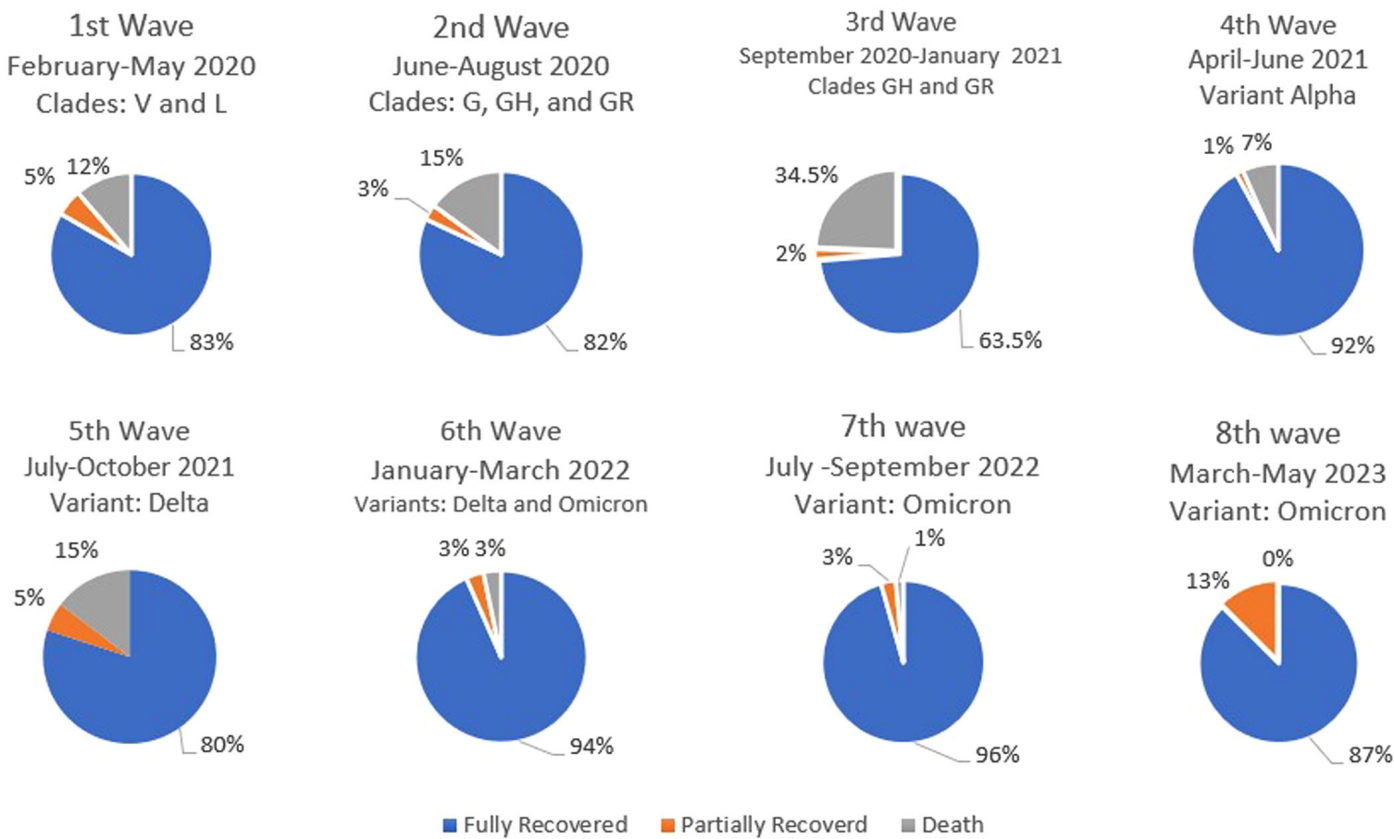
The distribution of mortality outcomes during the eight waves of the COVID‐19 pandemic in Iran. The classification of pandemic waves and identification of dominant circulating SARS‐CoV‐2 variants were based on publicly available data from the World Health Organization (WHO) and GISAID using a combination of case surges and the emergence of new variants. (Data sources: WHO [https://data.who.int/dashboards/covid19/cases?m49=364&n=c], GISAID [https://gisaid.org/hcov19-variants/]).

The study examined the mortality rates of the participants. Out of the total population, 1668 individuals fully recovered, 80 patients experienced partial recovery, and unfortunately, 226 individuals died due to the disease. These deaths were attributed to COVID‐19 based on WHO classification criteria for COVID‐19 mortality [10]. It's important to note that while these patients had confirmed COVID‐19 infection, many had pre‐existing conditions that may have contributed to mortality. The most common comorbidities among deceased patients included cardiovascular disease, diabetes, and respiratory conditions. However, COVID‐19 was determined to be the primary or contributing cause of death in these cases based on clinical assessment and documentation in accordance with WHO guidelines for COVID‐19 mortality reporting. The mortality rates varied across the different waves, reaching a peak of 34.5% during the third wave and then decreasing to zero mortalities in the eighth wave (Figure 1).

### 3.4 Biomarkers and Pandemic Progression: Examining the Dynamic Changes in CPK Levels and Troponin Status

We conducted an analysis on the levels CPK and positivity rates of troponin, during different waves of the pandemic. Our findings indicated that there were significant differences in CPK levels among the waves (*p*‐value < 0.001), with the third wave having the highest CPK levels (median) while the eighth wave had the lowest. On the other hand, the first wave had the highest percentage of positive troponin results, while the eighth wave had no positive troponin cases. However, there were no significant differences in troponin positivity rates across the waves (*p*‐value < 0.135) (Table 1).

### 3.5 Age and Biomarkers: Assessing the Impact of Age on CPK and Troponin

Further analysis showed no significant differences in CPK and troponin test results among different age groups (considering the age of 65 as a cut‐off point), suggesting that age does not influence these biomarkers in COVID‐19 patients (CPK: *p*‐value < 0.46, troponin: *p*‐value < 0.36) (Table 2).

**Table 2:** Biomarkers findings among demographics and outcome status in COVID‐19 patients.

	Total	CPK			Positive troponin	
	Number	Mean (SD)	Median (IQR)	*p* value	Number (%)	*p* value
Age						
< 64	1117	193.25 (377.55)	102.0 (126.5)	< 0.46	18 (1.6)	< 0.36
> 65	858	192.55 (348.41)	100.0 (147.5)		21 (2.4)	
Gender						
Male	1046	248.45 (467.71)	123.0 (159.0)	< 0.001	26 (2.5)	0.83
Female	929	130.45 (172.66)	78.0 (93.0)		13 (1.4)	
Outcome						
Full recovery	1668	181.63 (374.24)	93.0 (113.0)	< 0.001	27 (1.6)	0.006
Partial recovery	80	215.5 (234.18)	126.0 (166.75)		5 (6.3)	
Death	226	268.93 (324.84)	179.5 (224.5)		7 (3.1)	

### 3.6 Gender and Biomarkers: Uncovering Gender‐Specific Differences in CPK and Troponin

An analysis was conducted to investigate the relationship between gender and biomarkers. The results of the test revealed that there were significant differences in CPK levels between men and women. Men had significantly higher CPK levels (*p*‐value < 0.001) than women. However, there was no significant difference in troponin positivity rates between the genders (*p*‐value = 0.83) (Table 2).

### 3.7 Outcome Groups and Biomarkers: Investigating the Relationship Between CPK, Troponin, and Patient Outcomes

In our study, we examined the connection between the levels and positivity rates of biomarkers and the outcomes of patients. We found noteworthy variations among the groups of outcomes concerning CPK levels (*p*‐value < 0.001). Particularly, the levels of CPK were remarkably higher in the nonsurvivor group as compared to the group of patients who fully recovered (adjusted *p*‐value < 0.0001). Additionally, we discovered that there were significant differences in troponin positivity rates between the outcome groups (*p*‐value = 0.006) (Table 2).

### 3.8 Overall Model Performance

**Figure 2:**
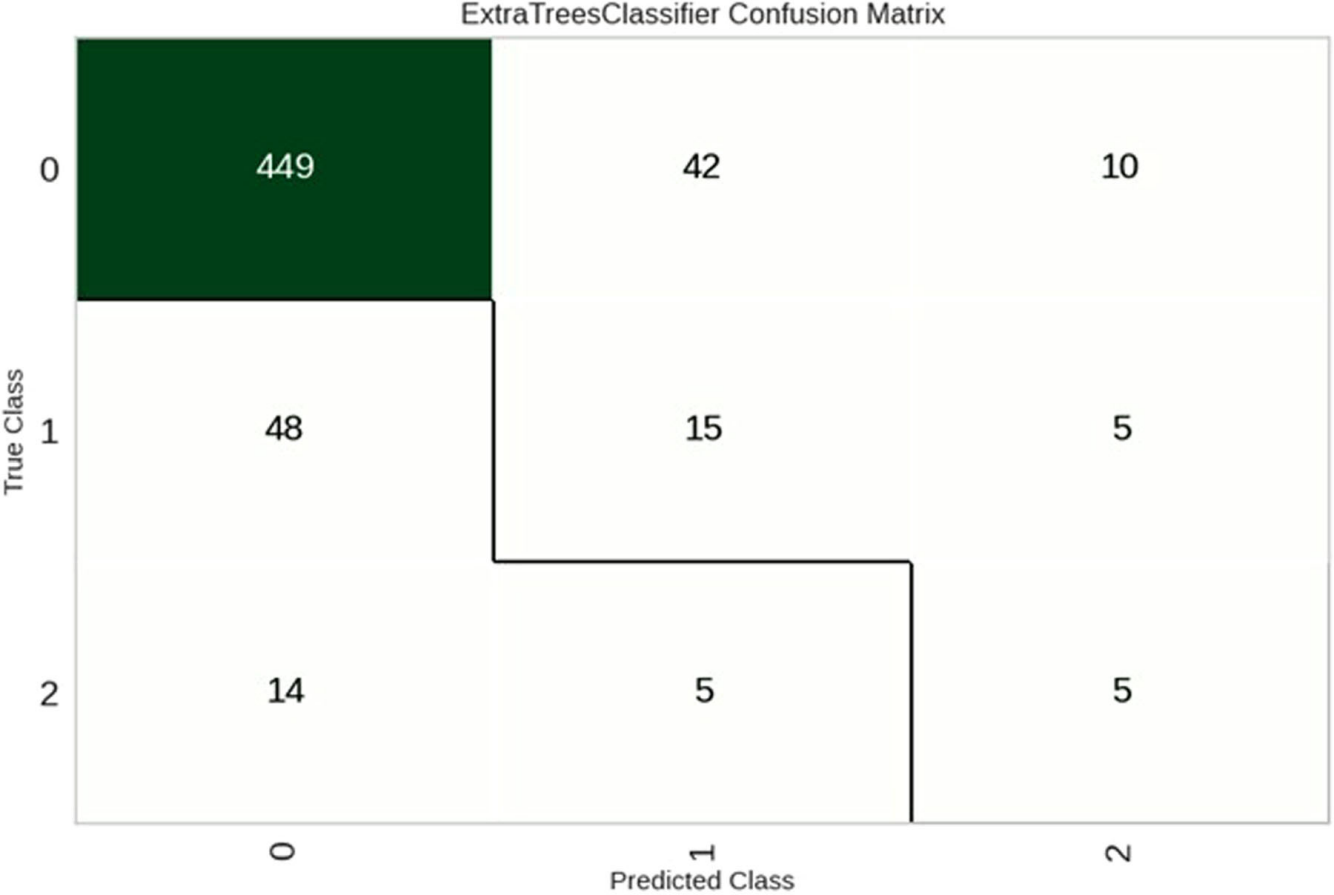
Confusion matrix: The confusion matrix illustrates the performance of our Extra Trees classifier across the three outcome classes. The diagonal cells represent correctly classified instances: 89.6% of “Full recovery” cases (Class 0) were correctly identified, while the model correctly classified only 22.0% of “Death” cases (Class 1) and 20.8% of “Partial recovery” cases (Class 2). This demonstrates the model's strong performance in identifying full recovery outcomes but reveals limitations in distinguishing between death and partial recovery outcomes. The off‐diagonal cells show misclassifications, with a notable number of Class 1 and Class 2 instances being incorrectly classified as full recovery, explaining the lower specificity (0.326) for the full recovery class.

**Figure 3:**
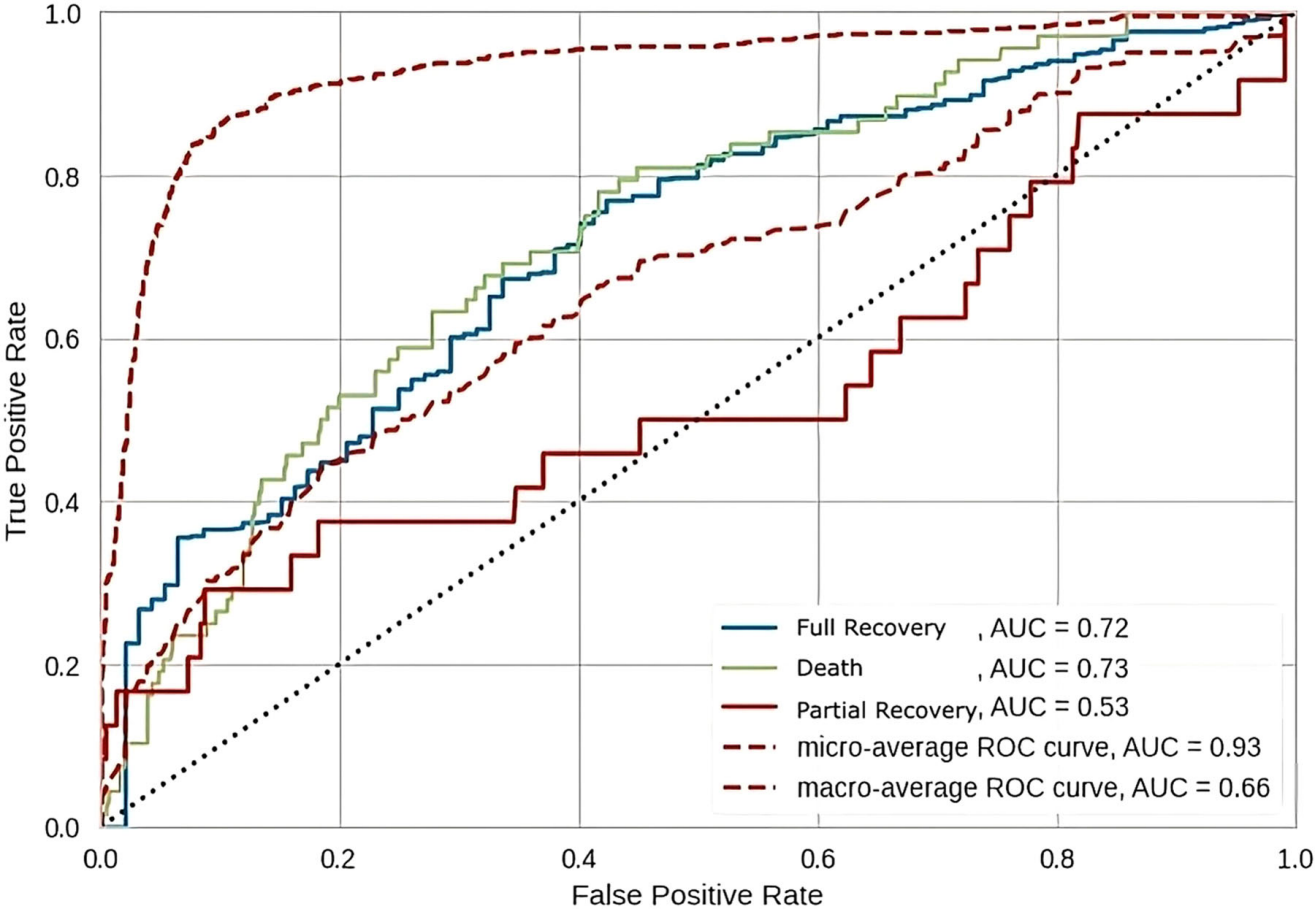
Receiver operating characteristic (ROC) curves for the ExtraTreesClassifier model. The figure shows the performance of the multi‐class classifier. Individual ROC curves are plotted for Class 0 indicating full recovery (blue, AUC = 0.68), Class 1 indicating death (green, AUC = 0.66), and Class 2 indicating partial recovery (solid red, AUC = 0.62). The overall model performance is assessed using two averaging methods: the micro‐average ROC curve (AUC = 0.90) and the macro‐average ROC curve (AUC = 0.65) shown with dashed lines. The dotted black line represents the baseline performance of a random classifier (AUC = 0.5).

The extra trees classifier had the most accuracy, and the overall accuracy of the model was 0.790, indicating that 79% of the instances were correctly classified. The AUC was 0.65, suggesting that the model's ability to discriminate between classes was moderate. The sensitivity was 0.790, meaning that the model correctly identified approximately 79% of the positive instances, while the specificity was 0.895, indicating that the model correctly identified nearly 90% of the negative instances. The confusion matrix and receiver‐operating characteristic curve (ROC) are represented in Figures 2 and 3, respectively.

### 3.9 Class‐Specific Model Performance

Table 3 provides a detailed view of the model's performance by presenting class‐specific evaluation metrics. For the “full recovery” class, the model achieved an accuracy of 0.807, an F1 score of 0.887, an AUC of 0.68, a sensitivity of 0.896, and a specificity of 0.326. The high sensitivity suggests that the model effectively identified instances of full recovery, while the lower specificity indicates that it may have misclassified some instances from other classes as full recovery. In the “death” class, the model had an accuracy of 0.831, an F1 score of 0.230, an AUC of 0.66, a sensitivity of 0.220, and a specificity of 0.910. The high specificity suggests that the model correctly identified a large proportion of non‐death instances, but the low sensitivity indicates that it struggled to identify actual death cases. For the “partial recovery” class, the model achieved an accuracy of 0.942, an F1 score of 0.227, an AUC of 0.62, a sensitivity of 0.208, and a specificity of 0.973. The high accuracy and specificity suggest that the model performed well in identifying instances that were not partial recovery. However, the low F1 score and sensitivity indicate that the model had difficulty correctly identifying actual partial recovery cases.

**Table 3:** Extra trees classifier evaluation metrics.

Class‐specific metrics	Full recovery	Death	Partial recovery
Accuracy	0.80776	0.83137	**0.94266**
F1 score	**0.88735**	0.23077	0.22727
AUC	**0.68**	0.66	0.62
Sensitivity	**0.89621**	0.22059	0.20833
Specificity	0.32609	0.91048	**0.97364**
PPV	**0.87867**	0.24194	0.25
**Overall metrics**			
Accuracy	0.790		
AUC	0.65		
Sensitivity	0.790		
Specificity	0.89545		

*Note:* Bold values indicate the highest score for each metric across the three classes.

Abbreviations: AUC, area under the curve; PPV, positive predictive value.

### 3.10 Comparative Analysis of Class Specific Metrics

**Figure 4:**
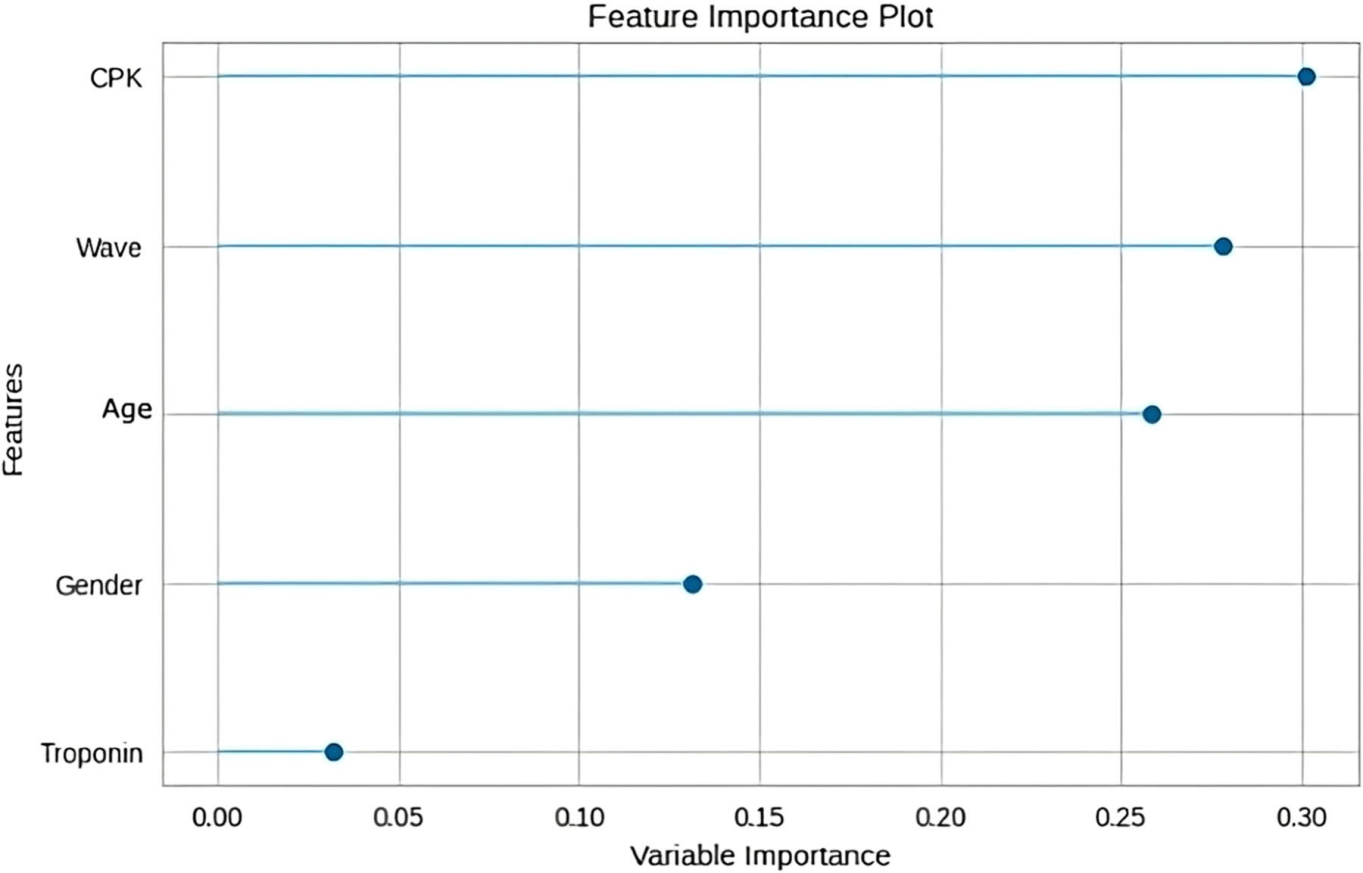
Variable importance plot: The variable importance plot ranks the predictive power of each feature in our model. CPK emerged as the most influential predictor of patient outcome, followed by COVID‐19 wave, patient age, and gender. Troponin status demonstrated the least predictive importance.

Comparing the class‐specific metrics, it is evident that the model performed best in identifying instances of full recovery, as indicated by the high sensitivity and F1 score. The model struggled more with the “death” and “partial recovery” classes, as evidenced by the lower sensitivity and F1 scores. As depicted in Figure 4, the most important predictor of patient outcome was CPK, followed by COVID‐19 wave, age, and gender, while troponin status had the least importance in forecasting the patient's prognosis.

## 4 Discussion

Since its initial outbreak in Wuhan in 2019, COVID‐19 has rapidly spread around the world, affecting different countries in several waves. Up until now, Iran has experienced eight waves of the virus caused by different strains of SARS‐CoV‐2. These strains have emerged due to multiple mutations in the virus's genome. Although SARS‐CoV‐2 has fewer mutations than most RNA viruses, it still undergoes mutations to evade the immune response, develop vaccine resistance, and adapt to its host. The predominant strains identified in Iran included B.1.1.7 (Alpha), B.1.617.2 (Delta), and B.1.1.529 (Omicron) [11]. CPK and troponin are two potential markers for organ damage that have gained significant attention for their association with cardiac and muscular injury. Studies have shown that COVID‐19 patients often have elevated levels of CPK and troponin, indicating their potential usefulness as biomarkers for disease severity [12, 13]. However, it is not yet clear how CPK and troponin test results change during different waves of the pandemic. Investigating how age, sex, and hospital outcomes vary during these waves can provide valuable insights into how COVID‐19 impacts different demographic groups. Additionally, analyzing the correlation between cardiac biomarkers and demographic variations can help us better understand the disease.

Our study found that there was a significant change in CPK levels during different waves of COVID‐19, while troponin status remained consistent throughout. The observed variations in CPK levels across different waves coincided with changes in several factors. Our data showed significant demographic shifts, with the mean age being highest during the third wave (61.36 ± 20.97 years) and lowest during the seventh wave (48.36 ± 24.31 years). Additionally, the male‐to‐female ratio changed from 1.36 in the third wave to 0.74 in the seventh wave. While our study cannot establish direct causation, these demographic changes, along with the emergence of different viral variants and increasing vaccination coverage over time, may have contributed to the observed trends in CPK levels. On the other hand, the consistency in troponin status suggests that it is more likely to change due to cardiac damage, particularly myocardial infarction, and is less affected by viral infection or other characteristics of each wave.

After examining the mortality rate among the SARS‐CoV‐2 waves, it was reported that the third wave was the most severe. This wave spread from Western countries, Australia, and Canada to other countries and was created by the B.1.1.413 variant. Upon closer examination of the CPK levels in each wave, it was observed that the third wave exhibited the highest median CPK levels, while the eighth wave had the lowest. This decrease in muscular damage may be attributable to multiple factors, including increased vaccine coverage during the later waves, the changes in circulating SARS‐CoV‐2 variants, and a demographic shift toward younger individuals [14]. Over 100 million COVID‐19 vaccine have been administered in Iran, with Sinopharm being the most commonly vaccine administered [15, 16]. It is important to note that Sinopharm uses traditional inactivated virus vaccine technology, which differs from the mRNA or viral vector platforms used in some other COVID‐19 vaccines. This distinction is relevant when interpreting our cardiac biomarker findings, as some studies have suggested different cardiac effect profiles between traditional and genetic technology‐based vaccines [17]. While the powerful role of vaccination in controlling the COVID‐19 pandemic is undeniable, our study did not specifically investigate the correlation between vaccination status and reductions in these biomarkers. Therefore, further research is needed to clarify the relative contributions of vaccination, viral evolution, and demographic changes to the observed trends in muscular damage.

During the third wave of COVID‐19, higher levels of CPK were observed. This could be because there were more older patients who may have had underlying health conditions, which led to more muscular damage. Our study also found that the mean age was highest during the third wave [18]. Age‐related factors and underlying health conditions are known to affect the severity of COVID‐19 and can cause damage to organs [19].

It is crucial to take into account that different strains of SARS‐CoV‐2 can affect various organs differently, including muscular tissues. Past research has indicated that the features and indications of COVID‐19 can vary across different waves, possibly due to the emergence of new viral strains [20]. Although the severity of lung involvement may vary among strains, they can also have different impacts on other organs, such as the muscles.

Previous studies have shown that males have higher levels of CPK than females [21]. Similarly, we noticed that the male‐to‐female ratio was highest during the third wave and lowest during the later waves. This difference in gender distribution might explain the higher levels of CPK seen during the earlier waves.

A similar scenario may also contribute to the troponin status. The lower efficacy of vaccination coverage in influencing the troponin trend could be a result of the more aggressive impact of the later waves of COVID‐19 on myocardial tissue. It is possible that the viral variants circulating during these waves have a heightened propensity to cause cardiac damage, leading to troponin positivity rates even in vaccinated individuals. These findings indicate that the level of cardiac damage remained relatively consistent across different waves, which aligns with a previous study demonstrating consistent cardiac damage between the first and sixth waves [22]. However, there are several additional factors that could explain this consistency. Firstly, the thresholds used to define a positive troponin status are well‐established for acute coronary syndromes. However, in the context of COVID‐19, these thresholds may need to be adapted. Previous studies in the field of COVID‐19 have suggested that a value above the 99th percentile upper reference limit of a normal reference population is the recommended threshold for defining an increased cardiac troponin level indicative of myocardial injury [23].

Secondly, it is important to consider the timing of biomarker measurements, as it can play a critical role in determining the levels of these biomarkers. For example, troponin, which is commonly used as a marker of cardiac injury, typically exhibits a delayed release with peak concentration occurring around 19–24 h after the onset of cardiac injury. This specific timing of troponin release may contribute to the consistent status observed across different waves of COVID‐19.

The consistency observed in the troponin status aligns with our model's findings. Our model identified CPK as the most accurate predictor of patient outcomes, followed by the COVID‐19 wave, age, and gender. Conversely, the troponin status was found to have the least significance in forecasting the patient's prognosis. This suggests that while troponin status remained consistent, they did not play a substantial role in predicting patient outcomes as compared to CPK levels and other factors. We acknowledge that our model's performance metrics reveal important limitations for clinical application. While the overall accuracy (0.79) appears reasonable, the moderate AUC (0.65) and particularly the low sensitivities for death (0.220) and partial recovery (0.208) outcomes significantly constrain the model's utility as a predictive tool. These values indicate our model fails to identify approximately 78%–79% of patients who eventually died or experienced partial recovery—critical outcomes where early identification could potentially alter clinical management. This limitation likely stems from multiple factors: class imbalance despite SMOTE‐Tomek implementation, binary rather than continuous troponin values, and absence of additional relevant clinical parameters such as comorbidities and inflammatory markers. Future model improvements could incorporate continuous biomarker measurements, additional cardiac and inflammatory indicators, temporal data capturing disease progression, and validation with larger balanced datasets. Such enhancements would be necessary before this approach could reliably guide clinical decision‐making for identifying high‐risk COVID‐19 patients.

There are additional limitations of our study that should be acknowledged. Firstly, it was a retrospective study, which introduces certain potential limitations such as recall bias and incomplete data. The reliance on medical records and patients’ memory may have influenced the accuracy and completeness of the information gathered. Additionally, the retrospective design of the study may have resulted in variations in the timing of biomarker measurements relative to symptom onset. The timing of phlebotomies can significantly impact biomarker levels, and without a standardized protocol for timing, there may be variability in the measurements obtained. It is important to acknowledge a key methodological limitation in our comparison of CPK and troponin as predictive biomarkers. While CPK was measured as a continuous variable, troponin was assessed qualitatively as positive or negative, following standard hospital protocols. This difference in measurement scales necessitates careful interpretation of our comparative analyses. The binary classification of troponin may underestimate its full potential as a predictive biomarker, as it does not capture the granular variations in troponin levels that could provide additional prognostic information. Future studies employing quantitative troponin measurements, such as those obtained through ELISA testing, would enable more direct comparisons with CPK levels and potentially reveal additional insights into the relative predictive value of these biomarkers. Additionally, our study did not specifically exclude patients based on factors known to affect CPK levels, such as statin use, steroid treatment, or alcohol consumption. These factors could potentially confound the interpretation of CPK levels as markers of COVID‐19 severity. Additionally, we did not have baseline estimates of CPK levels or troponin status from individuals without SARS‐CoV‐2 infection for comparison. Furthermore, the prolonged duration of our study spanned across multiple waves of the pandemic, which may have introduced challenges in maintaining consistency in the laboratory assays used for measuring the biomarkers. Changes in laboratory protocols, reagents, or equipment over time could potentially impact the comparability of the biomarker measurements.

Despite these limitations, this study offers valuable insights into how different factors influence biomarkers during the pandemic and how these biomarkers affect the COVID‐19 disease. Our findings suggest that CPK is a more reliable marker for assessing COVID‐19 severity and outcomes compared to troponin, particularly as the pandemic evolves with new viral strains. When interpreting clinical biomarkers, future studies should explore standardized protocols for biomarker measurement and consider the virus's evolving nature.

## Author Contributions

**Amirhossein Shahpar:** methodology, validation, writing – original draft. **Nazanin Zeinali Nezhad:** methodology, validation, writing – original draft. **Niloofar Farsiu:** writing – review and editing. **Marzieh Charostad:** writing – review and editing. **Masoud Rezaei:** formal analysis, software. **Faranak Salajegheh:** investigation. **Mohammad Pardeshenas:** data curation. **Seyedeh Mahdieh Khoshnazar:** resources. **Mohsen Nakhaie:** conceptualization, validation, project administration, supervision.

## Ethics Statement

Ethical approval for this study was obtained from the ethics committee (Code: IR.KMU.REC.1402.021).

## Consent

Informed consent was taken from each participant.

## Conflicts of Interest

The authors declare no conflicts of interest.

## Transparency Statement

The lead author Mohsen Nakhaie affirms that this manuscript is an honest, accurate, and transparent account of the study being reported; that no important aspects of the study have been omitted; and that any discrepancies from the study as planned (and, if relevant, registered) have been explained.

## Data Availability

All data analyzed for this study are included in the manuscript and tables.
